# Addressing the Socioemotional Dimension of Medical Education: Protocol for a Scoping Review of Student-Driven Development Strategies

**DOI:** 10.2196/66757

**Published:** 2025-07-24

**Authors:** Cintia Canato Martins, Marco Antônio Ribeiro Filho, Christian Guilherme Capobianco dos Santos, Luana Ribeiro Altrão lorino, João Daniel de Souza Menezes, Matheus Querino da Silva, Loiane Letícia dos Santos, Emerson Roberto dos Santos, Natalia Almeida de Arnaldo Silva Rodriguez Castro, Helena Landin Gonçalves Cristóvão, Randolfo dos Santos Júnior, Gerardo Maria de Araújo Filho, Patrícia da Silva Fucuta, Alba Regina de Abreu Lima, Vânia Maria Sabadoto Brienze, Denise Cristina Móz Vaz Oliani, Neide Aparecida Micelli Domingos, Maria Cristina de Oliveira Santos Miyazaki, Júlio César André

**Affiliations:** 1 Faculdade de Medicina de São José do Rio Preto São José do Rio Preto Brazil; 2 Faceres Medical School Faculdade Ceres São José do Rio Preto Brazil; 3 University Hospital Center Cova da Beira University of Beira Interior Covilhã Portugal

**Keywords:** medical education, socioemotional skill, competency development, scoping review protocol, medical students, curriculum development

## Abstract

**Background:**

The current international context, marked by complex global challenges, necessitates the rapid intervention of higher education institutions to contribute to social transformation. Medical education, in particular, is undergoing significant changes influenced by various factors, including the evolving health care environment and shifting social expectations. Socioemotional skills are increasingly recognized as essential competencies for medical professionals, contributing to better patient care, improved communication, and enhanced teamwork.

**Objective:**

This scoping review aims to provide a comprehensive overview of the current literature on strategies employed by medical students to develop and promote their socioemotional skills in academic and hospital settings.

**Methods:**

The review will be conducted according to the guidelines of the Joanna Briggs Institute, and the results will be presented in accordance with PRISMA-ScR (Preferred Reporting Items for Systematic reviews and Meta-Analyses extension for Scoping Reviews). Eligibility criteria include studies focusing on medical students at any stage of their education, addressing strategies for developing socioemotional skills in academic or clinical contexts. Information sources include Embase, Emerald, PsycNet, PubMed, Scopus, and Web of Science, with searches conducted from January 2000 to April 2025. Gray literature sources, including ProQuest Dissertations & Theses Global, Conference Proceedings Citation Index, and OpenGrey, will also be searched. Two independent reviewers will screen titles, abstracts, and full texts. Data will be extracted using a standardized form and synthesized through narrative synthesis and thematic analysis.

**Results:**

Results from our initial searches indicate a diverse range of literature addressing student-driven strategies for socioemotional skill development in medical education. The scoping review is progressing according to the following timeline. Protocol development and registration was completed in January 2025. Search strategy refinement was completed in February 2025. The initial database searching was expected to be completed in March or April 2025. Title and abstract screening are anticipated to be performed in May or June 2025. Full-text review and data extraction are anticipated to be performed in July to August 2025. Data synthesis and analysis are anticipated to be completed by September or October 2025. The manuscript is expected to be prepared and submitted by November or December 2025.

**Conclusions:**

The findings of this review may have significant implications for medical education curricula, informing the integration of socioemotional skill development alongside traditional medical knowledge and technical skills. Furthermore, the review may uncover gaps in the existing literature, highlighting areas that require further research and investigation. This scoping review will contribute to the growing body of knowledge on the importance of socioemotional skills in medical education.

**International Registered Report Identifier (IRRID):**

PRR1-10.2196/66757

## Introduction

In the rapidly evolving landscape of medical education, the development of socioemotional skills among medical students has emerged as a critical area of focus. This growing emphasis is reflected in the priorities of organizations such as the Association of American Medical Colleges [1] and the Accreditation Council for Graduate Medical Education [2], which increasingly recognize the importance of these skills in medical training. These skills, encompassing empathy, emotional intelligence, communication, resilience, and interpersonal effectiveness, are increasingly recognized as fundamental to providing high-quality patient care, fostering effective teamwork, and maintaining physician well-being [3,4]. As the health care environment becomes more complex and demanding, medical education must adapt to ensure that future physicians are equipped not only with clinical knowledge and technical proficiency but also with the socioemotional competencies necessary to navigate the multifaceted challenges of modern medical practice [5]. This scoping review aims to explore and synthesize the current literature on student-driven strategies for developing socioemotional skills in medical education, addressing a critical gap in our understanding of how medical students actively cultivate these essential competencies.

For the purposes of this review, we define socioemotional skills as a set of competencies that encompass the ability to recognize and manage emotions, develop care and concern for others, establish positive relationships, make responsible decisions, and handle challenging situations effectively [6]. This definition includes, but is not limited to, empathy, emotional intelligence, self-awareness, social awareness, relationship management, and responsible decision-making.

We define student-driven strategies as approaches, techniques, or practices that are primarily initiated, developed, or implemented by medical students themselves to enhance their socioemotional skills. These strategies may occur within or outside formal curricula and can include self-directed learning, peer-to-peer initiatives, extracurricular activities, or personal development practices [7]. For example, studies have explored the use of student-led support groups to address empathy, self-efficacy, and mental health stigma [8], and a scoping review shows the advantages of peer-mentoring programs, which reports academic (development of clinical confidence, skills in patient care, procedural skills, medical knowledge, interviewing and examination skills) and nonacademic (confidence, social support, peer relationships, and communication skills) benefits for both mentors and mentees [9].

Although our primary focus is on student-initiated approaches, this review also considers formal strategies that incorporate significant student feedback or input, enabling a comprehensive understanding of socioemotional skill development. This comprehensive approach allows us to examine the full spectrum of student engagement in socioemotional skill development, ranging from entirely self-directed activities to student-driven initiatives within formal educational settings. By including both formal and informal strategies, we aim to provide a nuanced understanding of how students actively participate in and shape their socioemotional skill development across various contexts in medical education. This inclusive approach will enable us to capture the diverse ways in which students engage with and contribute to their socioemotional learning, whether through self-directed efforts outside the curriculum or through active participation and modification of formal educational activities. By considering this broad range of strategies, we can better understand the interplay between institutional efforts and student-driven initiatives in fostering socioemotional competencies among future health care professionals. This review will examine the interplay between institutional efforts and student-led initiatives, analyzing how formal programs influence student strategies and how student feedback shapes institutional approaches. By considering this interaction, we can gain a deeper understanding of the socioemotional skill development in medical students.

The COVID-19 pandemic has further underscored the importance of this educational transformation, highlighting the need for flexible, resilient, and emotionally intelligent health care professionals [10,11]. Student-led strategies can play a crucial role in addressing the challenges posed by the crisis, promoting well-being, effective communication, and teamwork in high-pressure environments. In this context, understanding how medical students actively develop their socioemotional skills becomes even more crucial, particularly in terms of student-driven strategies that complement formal curricula.

This scoping review seeks to address this knowledge gap, providing insights that can inform more effective and student-centered approaches to socioemotional skill development in medical education. By identifying and categorizing the strategies used by students (objective 1), exploring the contexts in which these strategies are employed (objective 2), and examining the reported outcomes (objective 3), this review will provide a comprehensive overview of student-led approaches to socioemotional skill development. Furthermore, by identifying barriers and facilitators (objective 4) and highlighting gaps in the literature (objective 5), this review will inform future research and educational interventions.

The urgency during the COVID-19 pandemic forced a swift transition from the analog approach of conventional education—characterized by in-person, often paper-based, and physically interactive teaching methods—to a more digital model, even in contexts where the use of digital tools was less prevalent [12].

Despite the growing recognition of the importance of socioemotional skills in medical practice, there is a lack of comprehensive understanding of how medical students actively develop these crucial competencies. Although several reviews have examined formal curricula and interventions for socioemotional skill development in medical education [13,14], to our knowledge, no scoping review has specifically focused on student-driven strategies for developing these skills [15,16].

This scoping review aims to address this critical gap in understanding how medical students actively develop their socioemotional skills, complementing the formal curricula and institutional efforts to integrate these competencies into medical education. By exploring and synthesizing the current literature on student-driven strategies, this review seeks to provide insights that can inform more effective and student-centered approaches to socioemotional skill development in medical education.

The mental health of the students is of critical importance. Studies indicate a relationship between excessive use of social networks and higher levels of depression and anxiety in medical students [17]. Although data published on medical students’ mental health during the COVID-19 pandemic have been conflicting, early detection and intervention strategies must be implemented to help them navigate challenging periods and be better prepared for other large-scale crises [12]. The integration of research and training is at the core of professional training and medical education. This integration manifests through a multifaceted approach.

1. Constructive process: it involves actively building knowledge and skills through hands-on research experiences and critical analysis of scientific literature.
2. Contextualization: the integration ensures that research activities are relevant to clinical practice and aligned with current health care challenges.
3. Formal coordination: educational institutions implement structured programs and curricula that systematically incorporate research components throughout medical training.
4. Scientific thinking: the integration aims to cultivate analytical and investigative mindsets in medical students, encouraging evidence-based practice.
5. Dialectical relationships: this approach fosters a continuous dialogue between theoretical knowledge and practical application, as well as between established medical paradigms and emerging research findings.

Through this comprehensive integration, medical education seeks to produce physicians who are not only skilled practitioners but also critical thinkers capable of contributing to the advancement of medical science [18,19]. The integration of research and training in medical education can promote the development of socioemotional skills by encouraging students to reflect on their experiences, collaborate with others, and apply ethical principles in their practice. By participating in research projects, students can develop empathy, communication skills, and problem-solving abilities, which are essential for providing patient-centered care.

Consequently, medical training requires an intertwining where the performance of teachers and the adequate management of emotions by students will positively impact the values that should accompany future professionals [20,21]. This emotional management influences professional values in several key ways:

1. Empathy enhancement: effective emotional regulation fosters greater empathy, enabling future physicians to better understand and respond to patient needs and experiences [22].
2. Ethical decision-making: improved emotional awareness contributes to more nuanced ethical reasoning, crucial for navigating complex medical dilemmas [23].
3. Resilience building: emotional management skills bolster resilience, reinforcing values such as perseverance and adaptability in challenging health care environments [24].
4. Professionalism: emotional intelligence cultivated through proper emotion management underpins professional behaviors, including respectful communication and maintaining appropriate boundaries [25].
5. Cultural competence: enhanced emotional awareness facilitates greater cultural sensitivity, aligning with values of inclusivity and equitable care [26]. The adequate management of emotions by students will positively affect the values that should accompany future professionals. Emotional management can influence ethical decision-making, resilience, and cultural competence, enabling physicians to provide compassionate, equitable, and culturally sensitive care.

By developing these emotional competencies, medical students are better equipped to embody and sustain the core values essential to compassionate, ethical, and effective medical practice.

The primary objective of this scoping review is to provide a comprehensive overview of the current literature on strategies employed by medical students to develop and promote their socioemotional skills in academic and clinical settings. Although this review primarily focuses on strategies used in academic and clinical settings, it also acknowledges the relevance of experiences in broader medical contexts such as primary care, global health, telemedicine, and public health, as these experiences can significantly influence the development and application of socioemotional skills.

Specific objectives include the following.

1. Identify and categorize the various strategies used by medical students to develop socioemotional skills.
2. Explore the contexts in which these strategies are employed (eg, classroom, clinical rotations, extracurricular activities).
3. Examine any reported outcomes or effectiveness of these student-driven strategies.
4. Identify potential barriers and facilitators to student-driven socioemotional skill development.
5. Highlight gaps in the current literature and areas for future research.

The findings of this review have the potential to significantly impact medical education by (1) informing the design of more effective and student-centered approaches to socioemotional skill development in medical curricula, (2) identifying potential barriers and facilitators to skill acquisition from the student perspective, (3) highlighting informal learning opportunities that can be leveraged in formal educational settings, and (4) guiding future research on the effectiveness of various strategies for developing socioemotional skills.

By comprehensively examining this underexplored aspect of medical education, our scoping review will contribute to the growing body of evidence supporting the integration of socioemotional skill development in medical training, ultimately leading to better-prepared health care professionals and improved patient care.

## Methods

### Study Design

This scoping review protocol has been developed based on the methodological framework outlined by Arksey and O’Malley [13], further refined by Levac et al [14], and the Joanna Briggs Institute Manual for Evidence Synthesis [15]. This review will be reported in accordance with PRISMA-ScR (Preferred Reporting Items for Systematic reviews and Meta-Analyses extension for Scoping Reviews) [16]. The scoping review protocol was registered on the Open Science Framework.

### Review Question

The scoping review question was developed by applying the population, concept, and context framework [27,28]: what has been published about the strategies for developing or promoting (concept) socioemotional skills (context) in medical students (population)?

### Eligibility Criteria

Studies will be included if they meet the following criteria.

1. Population: medical students at any stage of their undergraduate or graduate medical education.
2. Concept: strategies, approaches, or methods used by students to develop or enhance socioemotional skills. These may include, but are not limited to, communication, empathy, emotional intelligence, teamwork, leadership, and self-awareness.
3. Context: academic settings (eg, classrooms, simulation centers) and clinical environments (eg, hospitals, clinics) where medical education takes place. This includes both formal educational activities and informal or extracurricular contexts where students engage in socioemotional skill development.
4. Study design: all types of primary research studies (qualitative, quantitative, and mixed methods), as well as review papers and gray literature (eg, theses, conference proceedings).
5. Time frame: studies published from January 2000 to the present, to capture contemporary approaches in medical education.
6. Language: studies published in English, Spanish, or Portuguese.

Studies will be excluded as follows.

1. Focus solely on formal curricular interventions implemented by faculty without student input or initiative.
2. Address only the development of clinical or technical skills without consideration of socioemotional competencies.
3. Involve health care professionals other than medical students (eg, nursing students, practicing physicians) as the primary population of interest.

The criteria mentioned above can be revised according to the available literature [13]. Although our primary interest lies in student-driven strategies, we recognize that these can occur within both formal and informal educational contexts. Therefore, our review will include studies that examine (1) entirely self-directed or informal activities initiated by students outside of the formal curriculum, (2) student-led or student-modified activities within formal educational settings, and (3) formal educational strategies that incorporate significant student input or allow for substantial student agency in skill development.

By including this range of contexts, we aim to capture the full spectrum of student engagement in socioemotional skill development. During the analysis phase, we will distinguish between these different types of strategies to provide a comprehensive understanding of how students actively participate in developing their socioemotional skills across various educational contexts.

### Types of Sources

The bibliographical research will be performed using the following databases: Embase, Emerald, PsycNet, PubMed, Scopus, and Web of Science. The search strategy was developed by a librarian expert in the subject and will be conducted using keywords in English, Spanish, and Portuguese to ensure comprehensive coverage of the literature. A comprehensive search strategy will be developed in consultation with a health sciences librarian. The strategy will include a combination of Medical Subject Headings (MeSH) terms and keywords related to medical students, socioemotional skills, and development strategies. The search strategy will be initially developed for MEDLINE and then adapted for other databases. A draft search strategy for MEDLINE is provided in Textbox 1.

**Search strategy.**

(“Medical Education” OR “Medical School*” OR “Medical Student*” OR “Medical Universit*” OR “Medical Facult*”) AND (“Competency-Based Education” OR “Competency Based Education” OR “Education, Competency-Based” OR “Competency-Based Educations” OR “Education, Competency Based” OR “Educations, Competency-Based” OR “Educación Basada en Competencias”) AND (“Socioemotional Skills” OR “Social Skills” OR “Interpersonal Skills” OR “Emotional Intelligence” OR “Empathy” OR “Self-awareness” OR “Social Awareness” OR “Relationship Management” OR “Affective Skills” OR “Emotional Competence” OR “Social Competence” OR “Emotional Regulation” OR “Social-Emotional Learning” OR “SEL”) AND (“Student-driven” OR “Self-directed Learning” OR “Peer-to-peer Learning” OR “Student-initiated” OR “Extracurricular Activities” OR “Personal Development” OR “Student-led” OR “Self-regulated Learning” OR “Autonomous Learning” OR “Independent Learning” OR “Student Agency” OR “Student Empowerment”)

To ensure a comprehensive search that captures both socioemotional skills and student-driven strategies, we have expanded our search terms. For socioemotional skills, we have included terms such as emotional intelligence, empathy, self-awareness, social awareness, relationship management, and affective skills. To capture student-driven strategies, we have incorporated terms such as self-directed learning, peer-to-peer learning, student-initiated, extracurricular activities, and personal development. Additionally, we have refined our search to focus specifically on medical students by including terms such as medical education, medical school, and medical students instead of broader higher education terms.

Additionally, we will search for gray literature by using ProQuest Dissertations & Theses Global, Conference Proceedings Citation Index (via Web of Science), and OpenGrey. Literature analysis will begin after identifying suitable studies and removing duplicates. The search strategy will be adequately refined if the literature list is found to be inappropriate [15,27].

### Study Selection

All identified citations will be collected and uploaded to the online Rayyan platform following the search strategy [29], and duplicates will be removed. Titles and abstracts will be selected and evaluated by 2 independent reviewers according to the review inclusion criteria. Potentially relevant sources will be fully retrieved.

The full text of the selected citations will be assessed against the inclusion criteria by 2 independent reviewers. Reasons for excluding full texts that do not meet the inclusion criteria will be recorded and reported in the review. At any selection stage, disagreements between reviewers will be resolved through discussion or with the mediation of a third reviewer. The search results and the study inclusion process will be represented according to PRISMA-ScR guidelines [16,30]. The complete PRISMA-Protocols checklist is provided as Multimedia Appendix 1 to ensure transparency and reproducibility of our review protocol. The research team will discuss the data selection results and develop a data extraction table after reaching a consensus.

### Data Charting

The data extraction instrument will be decided a priori, developed, and used to extract data from the included studies [13,28,30]. A standardized data extraction form will be developed and piloted on a sample of 5-10 included studies. Two reviewers will independently extract data from each included study. Any discrepancies will be resolved through discussion or consultation with a third reviewer. The following data will be extracted from each included study.

1. Study characteristics (author, year, country, study design)
2. Population characteristics (sample size, stage of medical education)
3. Context (academic setting, clinical setting, or both)
4. Socioemotional skills addressed
5. Description of student-driven strategies or approaches
6. Reported outcomes or effectiveness (if available)
7. Barriers and facilitators to strategy implementation (if reported)
8. Key findings and conclusions

Multimedia Appendix 2 shows the data extraction instrument. During data extraction, the draft tool will be modified and revised if necessary. Extraction will be performed using a double-blind scheme [28,31].

### Data Analysis

The data will be aggregated into themes, qualitatively described, and may be graphically represented. The final decision on the means to represent the results will be made based on the findings after the screening is completed [13,15,30]. The thematic analysis will follow the 6-phase approach outlined by Braun and Clarke (2006) [32]: (1) familiarization with the data, (2) generating initial codes, (3) searching for themes, (4) reviewing themes, (5) defining and naming themes, and (6) producing the report. Two independent reviewers will conduct the initial coding using NVivo software (QSR International) to ensure consistency and rigor. The coding process will be inductive, allowing themes to emerge from the data rather than fitting into a pre-existing coding frame. However, given that this scoping review will analyze diverse types of data extracted from published studies—including study characteristics, population details, intervention descriptions, and reported outcomes—our thematic analysis will be adapted to focus on identifying overarching themes related to student-driven strategies for socioemotional skill development in medical education. Specifically, the initial coding will involve categorizing the extracted data based on the type of strategy employed (eg, self-directed learning, peer-to-peer initiatives), the context in which it is implemented (eg, academic, clinical), and the socioemotional skills targeted (eg, empathy, resilience). Subsequent theme development will focus on synthesizing these categories to identify common patterns, key components, and potential mechanisms underlying the effectiveness of different student-driven strategies. This approach will allow us to move beyond a mere summary of individual studies and generate a more holistic understanding of how medical students actively develop their socioemotional skills.

A codebook will be developed and refined throughout the analysis process, with clear definitions and examples for each code and theme. To enhance trustworthiness, we will employ member checking with a subset of the included studies’ authors and engage in ongoing critical reflection and discussion among the research team. The final thematic framework will be reviewed by all the team members to ensure that it accurately represents the data and addresses the research objectives. This is a scoping review, and a formal quality assessment of the included studies will not be conducted. However, we will provide a narrative summary of the overall nature and quality of the evidence base.

Our analysis will account for the diverse types of study designs and sources included in this scoping review. We will employ a stratified approach to manage and analyze different types of evidence: (1) primary research studies (quantitative, qualitative, and mixed methods) will be analyzed based on their methodological approach, key findings, and relevance to student-driven strategies for socioemotional skill development; (2) review papers will be examined for their synthesis of existing evidence, key themes, and gaps identified in the literature; (3) conference proceedings will be analyzed for the emerging trends, innovative approaches, and preliminary findings that may not yet be published in peer-reviewed journals; and (4) guidelines and recommendations from scientific societies will be assessed for their practical implications and alignment with student-driven approaches.

To manage potential overlap between sources, particularly between primary studies and reviews, we will cross-reference the included studies and note any instances where the same primary data are reported in multiple sources. In such cases, we will prioritize the most comprehensive or recent report of the data.

The double-blind scheme mentioned earlier refers to a process where 2 reviewers independently extract data from each included study. To elaborate on this process: (1) two reviewers will independently extract data from each included source by using the predetermined data extraction form; (2) the reviewers will be blinded to each other’s extractions to prevent bias; (3) after completing the independent extractions, the reviewers will compare their results; (4) any discrepancies in the extracted data will be discussed and resolved through consensus; and (5) if consensus cannot be reached, a third reviewer will be consulted to make a final decision. This rigorous approach ensures the accuracy and reliability of the data extraction process, minimizing potential bias and errors.

## Results

The scoping review is progressing according to the following timeline. Protocol development and registration was completed in January 2025. Search strategy refinement was completed in February 2025. The initial database searching was expected to be completed in March or April 2025. Title and abstract screening are anticipated to be performed in May or June 2025. Full-text review and data extraction are anticipated to be performed in July to August 2025. Data synthesis and analysis are anticipated to be completed by September or October 2025. The manuscript is expected to be prepared and submitted by November or December 2025. As of April 2025, we have completed the protocol development, search strategy refinement, and initiated the database searching. Preliminary results from our initial searches indicate a diverse range of literature addressing student-driven strategies for socioemotional skill development in medical education. The next steps involve screening titles and abstracts to identify relevant studies for full-text review. The final results, expected by December 2025, will include a comprehensive mapping of student-driven strategies, identifying gaps in current knowledge, and recommendations for future research and curriculum development in this field. In our final analysis and presentation of results, we will employ a multifaceted approach to illustrate the complex relationships among student-driven strategies, educational contexts, and outcomes.

1. Conceptual framework: we will develop and present a comprehensive conceptual framework that visually maps the interrelationships between the identified strategies, contexts, and reported outcomes.
2. Network analysis visualization: a network diagram will be created to demonstrate the connections and potential synergies between different strategies and their contexts.
3. Strategy-context matrix: we will construct a matrix cross-tabulating student-driven strategies against various contextual factors and reported outcomes, facilitating the identification of patterns and trends.
4. Temporal analysis charts: where longitudinal data are available, we will create timelines or trajectory maps to illustrate the evolution of strategies over the course of medical education.
5. Comparative strategy charts: we will develop comparative charts highlighting the relative strengths, limitations, and contextual dependencies of different student-driven strategies.
6. Thematic concept maps: using thematic analysis techniques, we will create concept maps illustrating key themes and subthemes emerging from the literature.

These analytical approaches will be complemented by a narrative synthesis to provide a rich, contextual understanding of the landscape of student-driven socioemotional skill development in medical education. This multidimensional analysis will facilitate the identification of best practices, knowledge gaps, and future research directions.

## Discussion

### Anticipated Significance

This scoping review will provide a comprehensive overview of student-driven strategies for developing socioemotional skills in medical education. By synthesizing the available evidence, we anticipate that this review will (1) inform medical educators and curriculum developers about effective student-centered approaches to socioemotional skill development, (2) highlight innovative strategies that could be incorporated into formal medical curricula, (3) identify potential barriers and facilitators to socioemotional skill development from the student perspective, and (4) guide future research by identifying gaps in the current literature and areas requiring further investigation.

### Potential Implications

The findings of this review may have significant implications for medical education.

1. Curriculum development: results may inform the integration of student-driven approaches into formal curricula, potentially enhancing the effectiveness of socioemotional skill training.
2. Student support: understanding student-initiated strategies can help educators better support and facilitate student efforts in developing these crucial skills.
3. Assessment methods: insights into student-driven approaches may inform the development of more authentic assessment methods for socioemotional competencies.
4. Educational policy: findings may influence educational policies related to the incorporation of socioemotional skill development in medical education standards and accreditation criteria.

### Strengths and Limitations

The strengths of this review include its comprehensive search strategy, inclusion of multiple languages, and focus on an underexplored aspect of socioemotional skill development in medical education. However, potential limitations include the possibility of missing relevant studies due to language restrictions and the challenge of synthesizing diverse types of evidence.

Socioemotional skills are increasingly recognized as essential competencies for medical professionals, as they contribute to better patient care, improved communication, and enhanced teamwork [33]. These skills encompass a wide range of abilities such as empathy, emotional intelligence, resilience, and interpersonal communication, which enable physicians to navigate the complex and emotionally demanding nature of their work [34,35].

Despite the growing awareness of the importance of socioemotional skills in medical education, there is limited research on the specific strategies employed by medical students to develop and promote these skills. This scoping review aims to address this gap by providing a comprehensive overview of the current literature on the various approaches used by medical students to cultivate their socioemotional competencies in academic and hospital settings.

### Conclusions

This scoping review will provide a comprehensive overview of student-driven strategies for developing socioemotional skills in medical education. By mapping the current literature and identifying gaps in knowledge, this review will contribute to the growing body of evidence on the importance of socioemotional competencies in medical practice and inform future educational interventions and research directions.

## Appendix

Multimedia Appendix 1 PRISMA-P (Preferred Reporting Items for Systematic review and Meta-Analysis-Protocols) checklist.

Multimedia Appendix 2 Data extraction instrument.

## Abbreviations

MeSH: Medical Subject Headings
PRISMA-ScR: Preferred Reporting Items for Systematic Reviews and Meta-Analyses extension for Scoping Reviews
