# Supplementary material for: Addressing the Socioemotional Dimension of Medical Education: Protocol for a Scoping Review of Student-Driven Development Strategies
Source: JMIR Res Protoc. 2025 Jul 24;14:e66757. doi: 10.2196/66757 (PMC12332453; doi:10.2196/66757)
Supplement: Multimedia Appendix 2 [file resprot_v14i1e66757_app2.docx]

## Appendix 2: Data Extraction Instrument

## Data Extraction Instrument: Student-Driven Socio-Emotional Skill Development in Medical Education

**Study ID:** [Unique identifier assigned to each study]

**I. Study Characteristics:**

1. **Author(s):**
2. **Year of Publication:**
3. **Country of Study:**
4. **Study Design:**
   - Qualitative
   - Quantitative
   - Mixed Methods
   - Review
   - Other (Specify):

**II. Population Characteristics:**

1. **Sample Size:**
2. **Stage of Medical Education:**
   - Pre-clinical
   - Clinical
   - Internship/Residency
   - Other (Specify):

**III. Context:**

1. **Setting:**
   - Academic (e.g., classroom, simulation center)
   - Clinical (e.g., hospital, clinic)
   - Both

**IV. Socio-Emotional Skills Addressed:**

1. **List of Skills:** (e.g., communication, empathy, emotional intelligence, teamwork, leadership, self-awareness, resilience, stress management)

**V. Student-Driven Strategies/Approaches:**

1. **Detailed Description of Each Strategy:**
2. **Categorization of Strategies:** (e.g., peer-to-peer learning, reflective practice, extracurricular involvement, online resources, mentorship)

**VI. Reported Outcomes/Effectiveness:**

1. **Outcomes Measured:** (e.g., self-reported skills, observed behaviors, standardized assessments, academic performance)
2. **Effectiveness of Strategies:**
   - Positive effects reported
   - Mixed effects reported
   - No effects reported
   - Effectiveness not assessed

**VII. Barriers and Facilitators:**

1. **Barriers to Implementation:** (e.g., time constraints, lack of faculty support, curriculum limitations)
2. **Facilitators to Implementation:** (e.g., peer support, institutional resources, dedicated time for reflection)

**VIII. Key Findings and Conclusions:**

1. **Main Findings Related to Student-Driven Strategies:**
2. **Authors' Conclusions and Recommendations:**

**IX. Notes:**

[Space for any additional relevant information or observations]

Source: Author.
